# Regulation of arousal and performance of a healthy non-human primate using closed-loop central thalamic deep brain stimulation

**DOI:** 10.1109/NER52421.2023.10123754

**Published:** 2023-05-19

**Authors:** Jonathan L. Baker, Robert Toth, Alceste Deli, Mayela Zamora, John E. Fleming, Moaad Benjaber, Dana Goerzen, Jae-Wook Ryou, Keith P. Purpura, Nicholas D. Schiff, Timothy Denison

**Affiliations:** 1 Feil Family Brain and Mind Research Institute, Weill Cornell Medicine, New York, NY 10065, USA; 2 MRC Brain Network Dynamics Unit, Nuffield Department of Clinical Neurosciences, University of Oxford, OX1 3TH, UK; 3 Department of Neurosurgery, John Radcliffe Hospital, University of Oxford, Oxford OX3 9DU, UK; 4 Institute of Biomedical Engineering, Department of Engineering Science, University of Oxford, Oxford OX3 7DQ, UK

**Keywords:** Arousal Regulation, Central Thalamus, Closed-loop, Deep Brain Stimulation

## Abstract

Application of closed-loop approaches in systems neuroscience and brain-computer interfaces holds great promise for revolutionizing our understanding of the brain and for developing novel neuromodulation strategies to restore lost function. The anterior forebrain mesocircuit (AFM) of the mammalian brain is hypothesized to underlie arousal regulation of the cortex and striatum, and support cognitive functions during wakefulness. Dysfunction of arousal regulation is hypothesized to contribute to cognitive dysfunctions in various neurological disorders, and most prominently in patients following traumatic brain injury (TBI). Several clinical studies have explored the use of daily central thalamic deep brain stimulation (CT-DBS) within the AFM to restore consciousness and executive attention in TBI patients. In this study, we explored the use of closed-loop CT-DBS in order to episodically regulate arousal of the AFM of a healthy non-human primate (NHP) with the goal of restoring behavioral performance. We used pupillometry and near real-time analysis of ECoG signals to episodically initiate closed-loop CT-DBS and here we report on our ability to enhance arousal and restore the animal's performance. The initial computer based approach was then experimentally validated using a customized clinical-grade DBS device, the DyNeuMo-X, a bi-directional research platform used for rapidly testing closed-loop DBS. The successful implementation of the DyNeuMo-X in a healthy NHP supports ongoing clinical trials employing the internal DyNeuMo system (NCT05437393, NCT05197816) and our goal of developing and accelerating the deployment of novel neuromodulation approaches to treat cognitive dysfunction in patients with structural brain injuries and other etiologies.

## I Introduction

Application of closed-loop approaches in systems neuroscience and brain-computer interfaces hold great promise for revolutionizing our understanding of the brain and for developing novel neuromodulation strategies to restore lost function. The anterior forebrain mesocircuit (AFM) is hypothesized to underlie arousal regulation of the brain and striatum to support cognitive functions during wakefulness [1]. Dysfunction of arousal regulation is hypothesized to contribute to persistent deficits in executive attention, working memory, and chronic mental fatigue experienced in a variety of neurological disorders, and most prominently in patients following traumatic brain injury (TBI). Previously, we showed that central thalamic DBS (CT-DBS) produced robust regulation of daytime arousal and led to the recovery of cognitive and motor function in a patient who had been in a minimally conscious state for six years following a TBI [2]. In a recently completed clinical feasibility study (NCT02881151), we demonstrated that daytime CT-DBS restored executive attention and working memory in five moderate to severe TBI (msTBI) patients, and also reduced daytime mental fatigue in two patients [3]. Despite these promising clinical results, our current mechanistic understanding of arousal regulation is limited by our inability to chronically record, analyze, and modulate the state of arousal in unrestrained subjects. In prior animal studies [4] [5] we showed that CT-DBS in healthy non-human primates (NHP) can markedly shift activity patterns across the AFM and robustly facilitate behavioral performance in visuomotor reaction time tasks. We’ve also demonstrated that complex high-order spectral ‘features’, extracted from global ECoG signals using modern machine learning (ML) methods, can be used to accurately predict decrements in NHP performance and the putative onset of ‘mental fatigue’ [6]. In the initial phase of this new study, we used pupillometry and *near* real-time analysis of prefrontal and premotor ECoG signals to initiate brief periods of CT-DBS under experimenter control in order to increase cortical-striatal arousal and to restore behavioral performance in one adult NHP. These proof-of-concept results directly informed the design and algorithmic tuning of a fully automated closed-loop DBS setup for the same animal. The automated experiment made use of customized clinical-grade DBS device, the DyNeuMo-X [7] to create a bidirectional closed-loop research platform, capable of detecting fluctuations in arousal in *actual* real-time. The DyNeuMo-X system was trained to classify cortical ECoG and thalamic LFP signals, and successfully initiated brief periods of CT-DBS that increased arousal and behavioral performance. The implementation and testing of the DyNeuMo-X in a healthy non-human primate (NHP) subject supports our ongoing clinical studies using the internal DyNeuMo system: CADET for pediatric epilepsy (NCT05437393); MINDS for multiple system atrophy (NCT05197816). Our NHP model allows for the development and de-risking of clinically desirable stimulation algorithms, thereby reducing closed-loop algorithms to practice in a fully implantable system that can be deployed in a variety of conditions. Overall, these results represent a critical first step toward developing and accelerating the deployment of novel neuromodulation approaches to effectively treat cognitive dysfunction in patients with structural brain injuries and other etiologies.

## II Methods

### A Behavioral Task and Experimental Setup

**Fig. 1:**
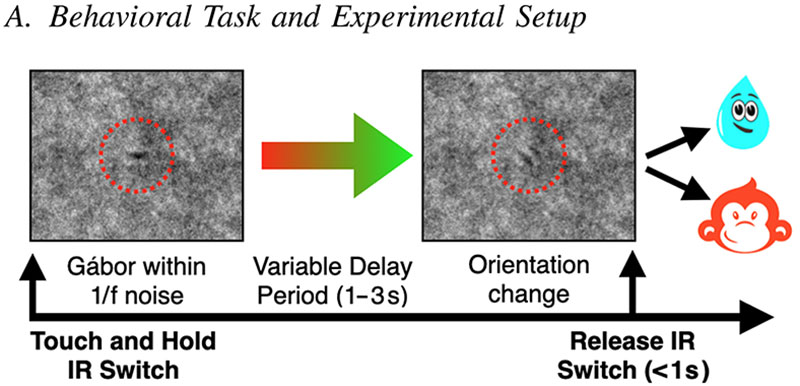
Structure of the behavioral task

All animal work was carried out in strict accordance with the NIH Guidelines for Use of Animals in Research, under a protocol reviewed by the Weill Cornell Medical College Institutional Animal Care and Use Committee (IACUC). Animals were cared for by the Research Animal Resource Center (RARC) at Weill Cornell Medicine. The animal was trained to perform a visuomotor reaction time task for liquid rewards Fig. 1. The animal initiated trials by touching and holding an infrared (IR) switch (Crist Instruments Company Inc., Hagerstown, MD, USA) linked to custom behavioral task programs designed in MonkeyLogic [8] that controlled the visual appearance of a central gray-scale Gábor-patch (3° of visual angle) embedded in a 1/f noise pattern on an LCD screen. The animal had to maintain fixation on the Gábor within an invisible constraint window (dotted red circle, 4–6°) as the spatial phase of the Gábor changed (every 300 ms) for a variable number of phase transitions (lasting 1–3 s) before changing orientation (from 0° to 22.5–90°). The change in orientation was the ‘GO’ cue and the animal had to remove its hand from the IR switch within 1 s to receive a liquid reward. Experimental sessions typically lasted 1–3 h until the animal was satiated. Horizontal and vertical eye movements, along with pupil diameter were recorded using an Oculomatic Pro v1.9.7 (Neuro-Software Developers Inc., Jacksonville, FL, USA) eye tracking system. A custom circuit that included a vibration sensor (801S) was attached to the animal’s behavioral chair to detect body movements. Broadband neural signals were recorded with an RZ2-4 data acquisition system (TDT – Tucker Davis Technologies Inc., Alachua, FL, USA) at a sampling rate of 25 kHz running TDT’s Synapse software. Synapse was also used to synchronize the behavioral task data, body movements and eye signals to the electrophysiological data.

### B NHP Cephalic Implant

**Fig. 2:**
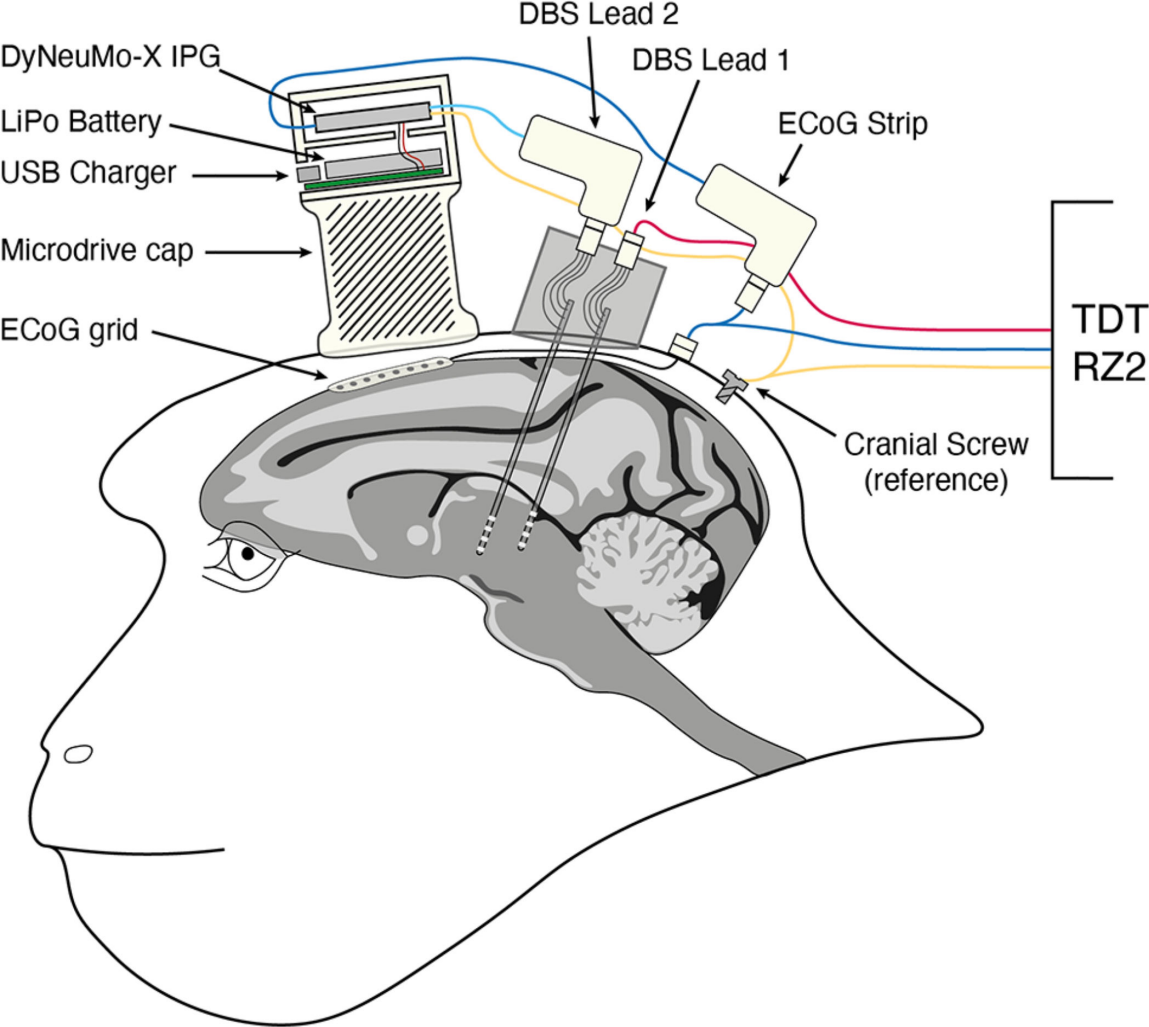
NHP cephalic implant and DyNeuMo-X setup. The externalized IPG was connected to DBS lead 2, and four of the eight ECoG channels. The remaining ECoG channels, as well as DBS lead 1 were connected to the RZ2 system for reference measurements.

The animal was chronically implanted with two NHP-scaled (0.85 μm OD) directionally-segmented DBS leads (Heraeus Medical Components) to target rostral and caudal portions of the central lateral (CL) nucleus of the right central thalamus. Each DBS lead consisted of four equally spaced rings (0.5 μm); each ring had three equally sized contacts (0.5 μm). We estimated the locations of the two DBS leads using previously published methods [5], and in this animal, the upper two rings of contacts for both DBS leads were positioned within the CL nucleus. The lower two rings were located within the lateral portion of the medial dorsal (MD) and the centralmedian (CM) nuclei. Following implantation and recovery, a monopolar stimulation review (0–3mA, 200 Hz) for all 12 contacts and each three-contact ring was conducted. The behavioral effects met our expectations for DBS with contacts located in the central thalamus: stimulation of contacts within CL produced consistent pupil dilation with currents as low as (0.25mA and consistent akathisia at (2–3mA; stimulation of contacts within MD at (1.0mA and above produced consistent leftward conjugate eye movements; stimulation within CM at (1.0mA and above produced consistent left hand and left arm movements and postural adjustments. Based on this monopolar review, stimulation was limited here to the upper two rings of contacts. The NHP was also instrumented to allow for the recording of neurophysiological signals. A 128-microelectrode microdrive (Gray Matter Research LLC, Bozeman, MT, USA) was implanted over the right prefrontal/premotor cortex to target multiple cortical and subcortical areas and an 8-channel epidural ECoG strip (Fetz-Spinal Cord 8, CorTec GmbH, Freiburg, Germany) was implanted adjacent to the midline, over prefrontal (areas 8B, 9L) and premotor (areas 6DC, 6DR) cortex. During the non-DBS experiments, we used the TDT RZ2 system to record from the ECoG strip, the two DBS leads and the 128-ch microdrive while the animal performed the behavioral task. During the closed-loop DBS experiments, a single differential signal was recorded from the rostral and caudal ECoG electrodes using a custom adapter connected to one lead of the DyNeuMo-X IPG Fig. 2.

### C Closed-loop Deep Brain Stimulation

#### 1) Near real-time computer aided Closed-Loop DBS

We used TDT’s runtime SynapseAPI to import two ECoG signals into Matlab (R2021a, Mathworks Inc., Natick, MA, USA) using TDT’s SDK library. With some customization (in particular to ‘mtspectrumc.m’), the Chronux toolbox [9] provided efficient multi-taper estimates of the power spectral density (PSD) of the ECoG signals, enabling fast runtime visualization. We produced PSD estimates of 1 s-long non-overlapping segments of the ECoG signals in order to visualize them in a colored pile plot, along with a 60 s average PSD. This processing pipeline achieved a delay of approximately 1–2 s between data import and PSD visualization. Based on prior pupillometry results (Fig. 4) we focused the PSD analysis on the two ECoG electrodes located over the premotor (area 6DC) and prefrontal (area 9L) cortices. A 4-channel stimulator (STG4004, Multi Channel Systems GmbH, Reutlingen, Germany) with a compliance of 120 V and a max stimulation amplitude of 3.2 mA was connected to the DBS leads to provide independent current controlled stimulation. The stimulation waveform consisted of an 80 μs cathodic pulse with an isoelectric period of 60 μs followed by a 400 μs anodic pulse. During this set of DBS experiments, the stimulator was connected to DBS Lead 2 and bipolar stimulation was conducted between the upper two rings of contacts, at 1.5–2 mA, 200 Hz for 2 s when power within the ‘alpha-band’ (8–12 Hz) in the ECoG signals exceeded an experimentally derived threshold, which was plotted as a black line in the colored pile plot. When the threshold was exceeded, a brief auditory signal was used to alert the experimenter. The actual timing of stimulation therefore depended on the reaction time of the experimenter to the auditory signal.

#### 2) Real-time Closed-Loop DBS with the DyNeuMo-X research platform

To remove the experimenter from the loop, real-time closed-loop experiments were carried out using the setup shown in Fig. 2. The DyNeuMo-X IPG was fitted atop the 128-microelectrode drive and connected to DBS lead 2 to provide stimulation and to record from the the ECoG strip using custom connectors. DBS lead 1 was connected to the RZ2 to record local field potential (LFP) activity in order to validate changes in the DyNeuMo-X IPG output and to synchronize the output of the IPG with the RZ2 data streams. The externalized IPG was set to deliver current-mode stimulation within the upper two rings of contacts in DBS lead 2, with two pre-defined stimulation programs (P1, P2) that differed only in current magnitude. Stimulation was delivered in a bipolar biphasic pattern with equal 90 μs anodic and cathodic phases, and less than 10 μs inter-phase delay. The stimulation frequency was set to 125 Hz and the current magnitudes were 0.1mA for pattern P1, and 2.0 mA for pattern P2. The closed-loop algorithm would initiate transitions from the low-intensity baseline program P1 into the high-intensity P2 mode. Transitions between the programs were ramped at a rate of 0.01 mA/pulse, i.e. 1.25 mA/s to reduce paresthesia and possible startle responses. The stimulation state was locked for a duration of 3 s to prevent rapid switching. The contact impedances were typically in the range of 2–4 kΩ for each ring of three-contacts. The DyNeuMo-X IPG did not support simultaneous data-streaming while running in closed-loop mode.

## III Results

Our working hypothesis was that the onset of inattentive drowsiness in the NHP is associated with significant changes in the PSD recorded from the cortical surface (ECoG) and from the thalamus and if these changes could detected, in real-time, episodic CT-DBS could be used to arouse the brain and potentially restore performance. These changes included an increase in power within the ECoG theta- (4–7 Hz) and alpha- (8–12 Hz) bands, a decrease in high-frequency ECoG power (20 Hz <), and an increase in the broader ‘theta-alpha’ band power in the central thalamus. We hypothesize that these electrophysiological changes would start to appear as time-on-task and satiety increased. Repeated performance of simple tasks, when conducted over extended periods of time, requires sustained ‘mental effort’ resulting in increasing error rates and human subjects report that sustaining ‘mental effort’ over many trials leads to the subjective experience of ‘mental fatigue’ and accounts for the increasing error rate [10].

**Fig. 3:**
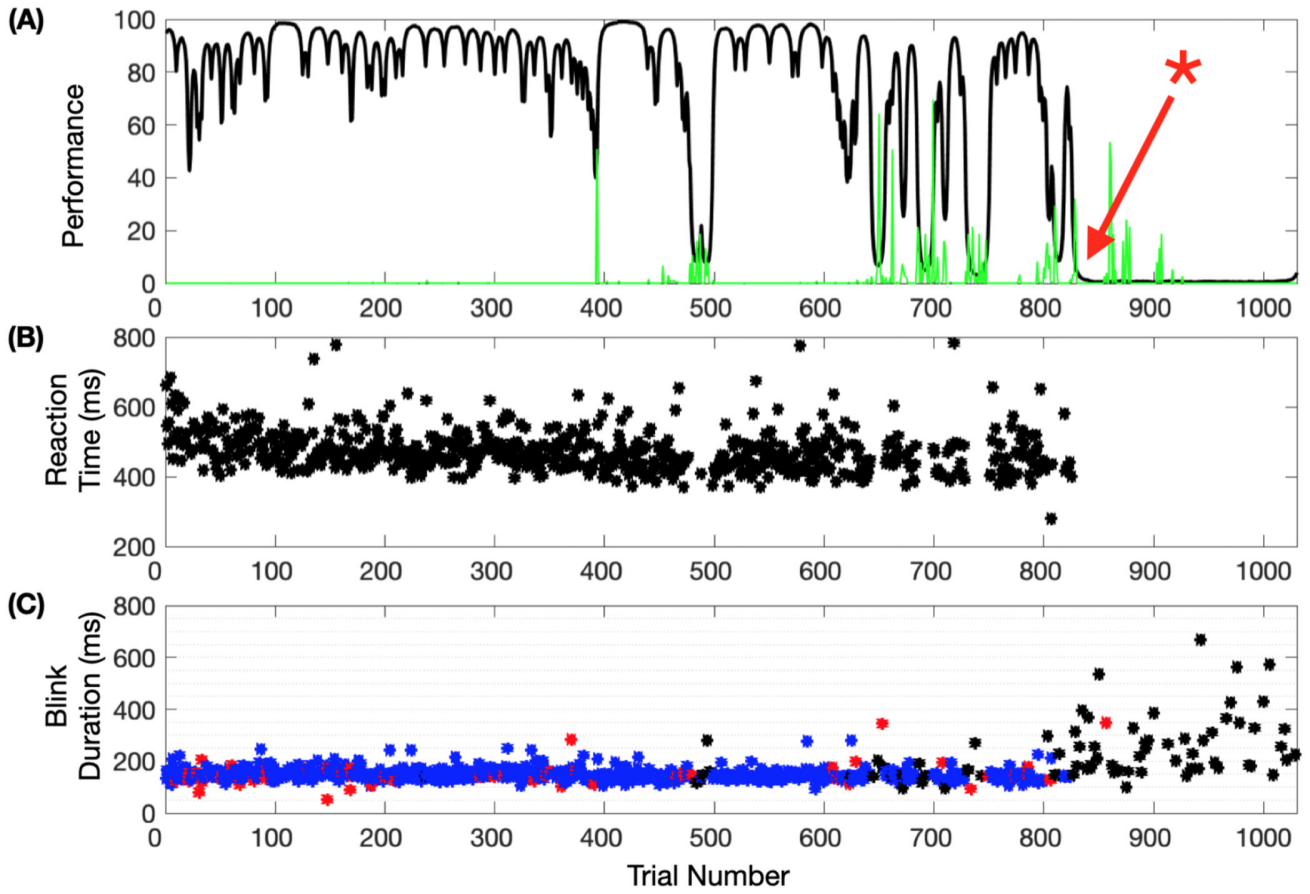
Task performance. (A) the animal’s performance estimate on the task across consecutive trials of an example session (black), along with body movements captured by the vibration sensor (green). The red asterisk marks when a CT-DBS experiment would typically begin. (B) Reaction times of correct trials. (C) Pupillometry events showing the time and duration of eye blinks. Blue and red blinks occurred during correct and incorrect trials, respectively. Blinks in black occurred when the animal was not engaged with the task. The first 1000 of 1800 trials are shown for clarity.

In order to test our hypothesis, we recorded cortical and thalamic LFP activity while the animal performed a vi-suomotor reaction time task over extended periods of time (Fig. 3), typically lasting 1–3 h. The animal usually performed well at the start of each experimental session and then, as anticipated, performance degraded as time on task increased. Decrements in the animal’s performance tended to correspond with increased body movements (as seen between trials 350 to 800) and significant increases in eye blink duration, as noted with the asterisk around trial 825, where the animal would then cease to engage with the task and stop performing. Based on this behavior, we analyzed blink durations in the context of correct and incorrect task performance and calculated the power spectral density (PSD) of 1 s segments of the 8-channel ECoG strip prior to blink onset, across 54 experimental sessions, using the multi-taper method [9]. An inter-blink interval less than 1 s was used to exclude overlapping data segments. We note two conditions under variable arousal levels for this animal: 1) when the NHP was engaged and performing well, blink durations averaged 148 ms (55–755 ms, n = 16 014); 2) when it was not engaged and/or performing incorrectly, the average blink duration was significantly increased to 246 ms (50–21 927 ms, n = 11 639) (Two-sample Kolmogorov-Smirnov test, p < 0.01).

**Fig. 4:**
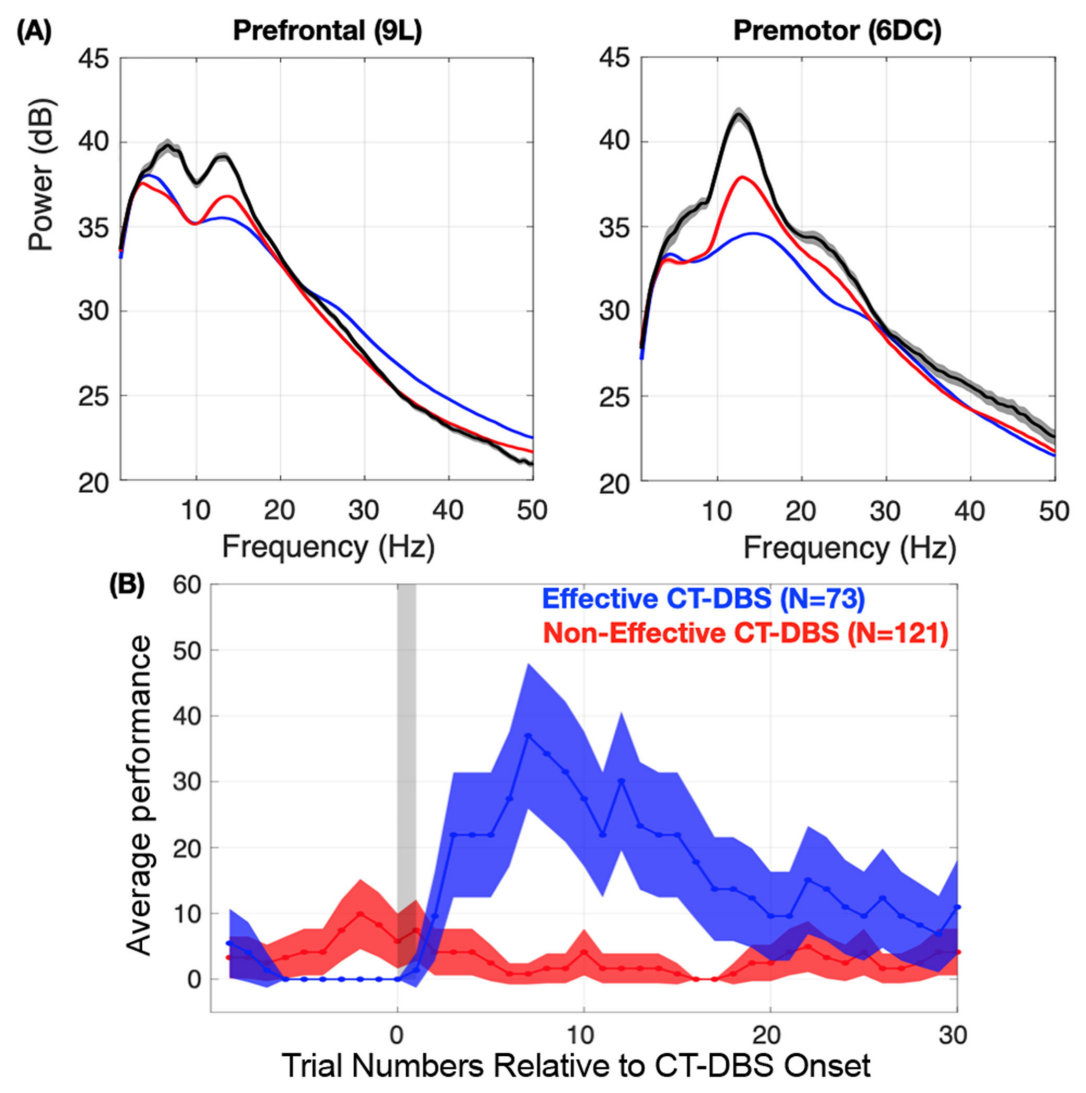
(A) Average power spectra density (PSD) of prefrontal (area 9L), left panel, and premotor (area 6DC), right panel, of one second segments of ECoG signals collected prior to the animal’s eye blinks. The blue traces represent the average PSD when the animal performed the task correctly. The red traces represent the average PSD when the animal performed the task incorrectly. The black traces represent the average PSD prior to eye closures greater than 1 second. Here we observe a shift in power in the alpha band as we move from blue, to red, to black traces (B) The animal’s average change in performance, relative to baseline performance, following a 2 second period of CT-DBS initiated by the experimenter (gray period). Stimulation periods that on average significantly enhanced performance are shown in blue, compared to stimulation periods when performance was not significantly enhanced.

We compared the PSD of the ECoG signals recorded under these two conditions, by first limiting the analysis to blink durations below 755 ms (red traces) and then separately for blink durations greater than 755 ms, which also contained full eye closures (black traces). As seen in Fig. 4A, oscillatory power within the ‘alpha-band’ was significantly increased during the incorrect task conditions (red and black traces), as compared to correct task conditions (blue traces). In Fig. 4A ECoG electrodes over the most rostral (area 9L) and caudal (area 6DC) are shown (both separated by 18 mm), however power in the ‘alpha-band’ range gradually increased rostral to caudal while the ‘theta-band’ power exhibited the opposite trend. This transient increase in the broad ‘theta-alpha-band’ power likely reflects a ‘state’ shift resulting from a reduction in synaptic inputs from central thalamic neurons to the frontal cortex [1].

To then test our working hypothesis we visualized, in near real-time, the PSD of the most rostral (area 9L) and caudal (area 6DC) ECoG electrodes in order to initiate brief 2s periods of continuous 200 Hz, 1.5–2.0 mA periods of CT-DBS, with the intention of electrically stimulating the central thalamus to enhance cortical arousal and possibly restore performance. A total of 194 CT-DBS periods, across six experimental sessions, were conducted and 73 of those periods resulted in a significant increase in performance relative to the pre-stimulation baseline period, as determined by an Odds-Ratio Test (p < 0.05). Interestingly, the relatively brief period of stimulation used had a long latency effect on performance, whereby the peak in performance occurred around 7 trials (8–10 s) following stimulation (Fig. 4).

**Fig. 5:**
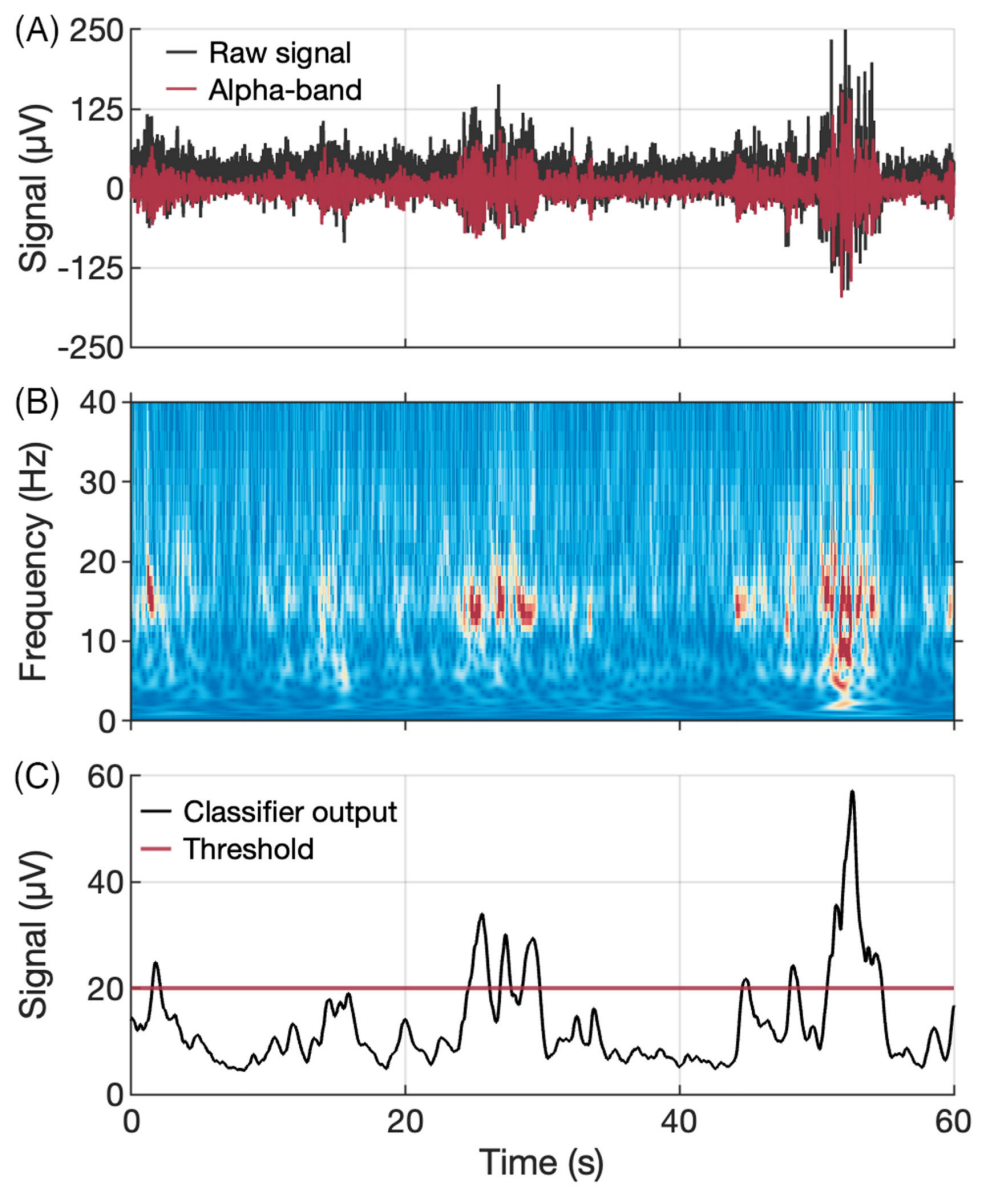
Example training data for closed-loop algorithm design. (A) Raw ECoG signal (black) recorded via the DyNeuMo-X, overlaid with 5–15 Hz ‘alpha-band’ activity (red) isolated offline. (B) Spectrogram of the raw ECoG signal of panel A. (C) Visualization of the final DyNeuMo-X adaptive algorithm output on the example data (black), and the selected classifier threshold (red).

**Fig. 6:**
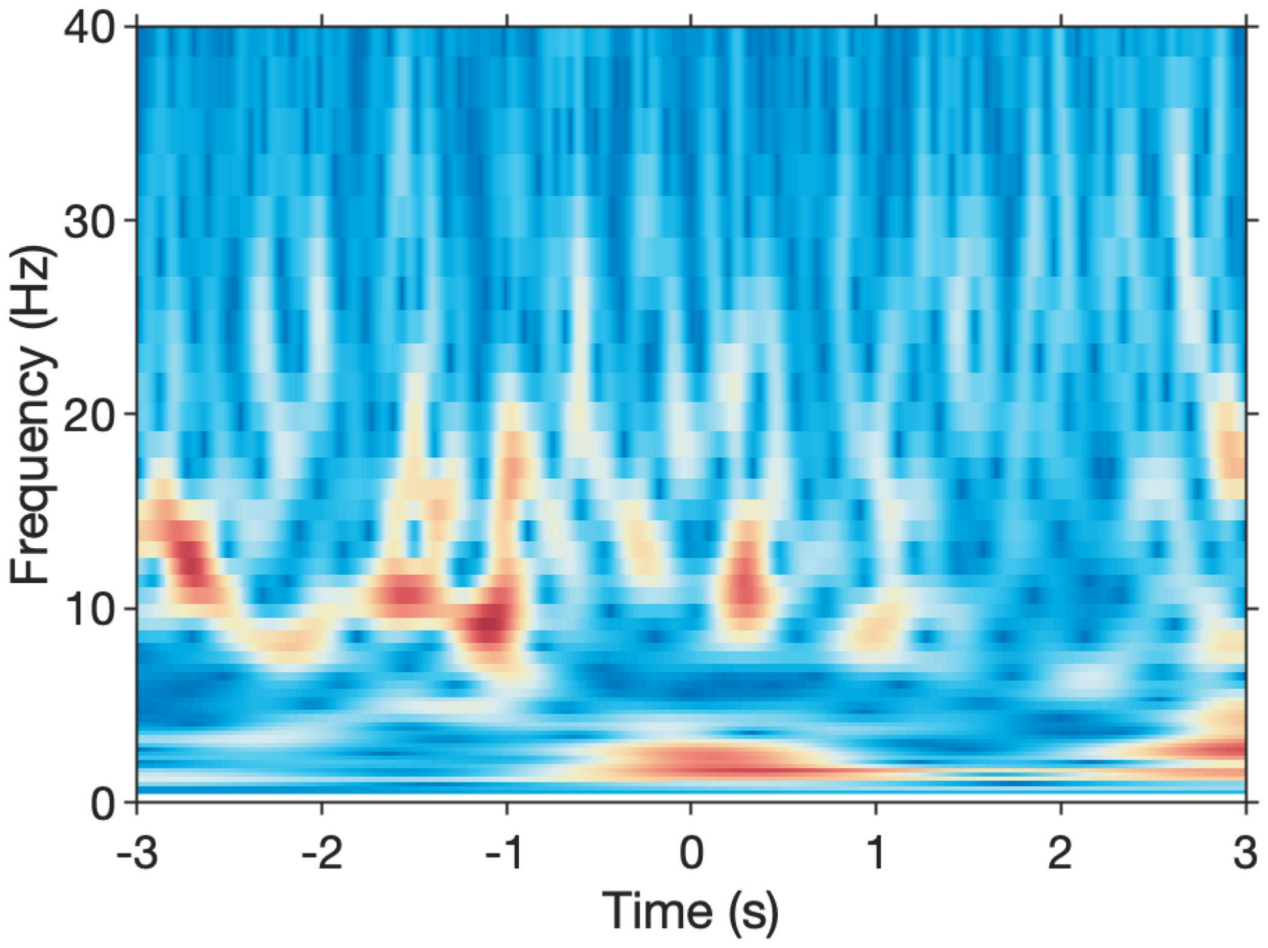
Triggered average (n = 36) of the continuous wavelet spectra of central thalamic LFP activity around cortical ‘alpha-band’ detector activation (P1 → P2 at t = 0), recorded from DBS lead 1 (caudal to the DyNeuMo-X lead). Events during motion were excluded.

We used the DyNeuMo-X IPG to validate this approach by first recording cortical ECoG activity when the animal was in a state of low motivation, i.e. not engaging with the behavioral task. Fig. 5A shows a 60 s sample of the raw ECoG recording overlaid with with activity in the alpha power passband (5–15 Hz). A spectrogram representation of the data (B) exhibits periods of markedly increased activity in the alpha-band that coincided with periods of inactivity and low motivation, and preceded by prolonged eye closures, one of which occurred around 53 s in Fig. 5B. We used this recording session as ‘training data’ to rapidly develop and tune a closed-loop classifier for ‘alpha-band’ activity. The state-classifier algorithm comprised the following computational blocks in order: a high-pass filter of 1.6 Hz cut-off (1st order), a band-pass filter for 5–15 Hz (4th order Butterworth), a rectifier stage, and finally a low-pass filter with 0.6 Hz cut-off (2nd order Butterworth). The classifier output for the ‘training signal’ is shown in Fig. 5C. A decision threshold of 20 μV was selected – exceeding this threshold would classify as a period of high ‘alpha-band’ activity, and initiate a P1 → P2 stimulation program transition, and vice versa. A debounce time of 2–3 s set the minimum stimulation duration, in order to be consistent with prior results. During the DyNeuMo-X closed-loop session, we simultaneously recorded LFP activity from the non-stimulated DBS Lead 1 using the RZ2 system. The non-stimulated lead also exhibited increased ‘alpha-band’ power prior to classifier activation, as shown in Fig. 6. Following the training sessions we used the DyNeuMo-X, while running the ‘alpha-band’ classifier, to toggle the amplitude of CT-DBS, from a baseline of 0.1 mA to 2.0 mA for several seconds, and in two separate experimental sessions the classifier consistently identified periods of increased ‘alpha-band’ power to initiate a change in stimulation. Unfortunately, bench marking the behavioral impact of the DyNeuMo-X closed-loop protocol to our in-house ‘human-in-the-loop’ sessions was not possible due to animal specific issues, however the testing of the DyNeuMo-X IPG and ‘alpha-band’ classifier is ongoing in additional animals.

## IV Discussion

Here we show that automated real-time detection of significant increases in ‘alpha-band’ oscillatory activity, recorded from ECoG electrodes placed over the prefrontal/premotor regions of a healthy NHP, can be used to initiate episodic CT-DBS, which directly enhances cortical arousal and was able to restore behavioral performance. Importantly, the increased ‘alpha-band’ activity, which was used to trigger CT-DBS, was measured when the animal’s eyes were fully open and just prior to lengthy eye blinks and/or full eye closures that likely reflect the onset of inattentive drowsiness. These results are consistent with humans subjects, where the on-set and subjective experience of ‘mental fatigue’ during the performance of cognitively demanding tasks, is significantly correlated with increased midline theta and alpha power [11]. The subjective experience of increasing ‘mental fatigue’ may reflect a generalized decrease in cortical arousal due to an overall decrease in the activity of neurons in the central thalamus, thereby resulting in a reduction in the background synaptic input to cortical and striatal circuits [1]. In this study, we stimulated within the central thalamus during these putative ‘mental fatigue’ periods in order to briefly enhance arousal of the brain, with the goal of restoring performance, as shown in Fig. 4. In a prior study, we showed that CT-DBS in healthy behaving NHPs can markedly shift the PSD profile of LFP activity recorded within regions of the prefrontal and premotor cortices and the dorsal striatum, and importantly, that these shifts LFP power were dependent on the amplitude and configuration of CT-DBS [4]. Mechanistically, electrical stimulation within the central thalamus is hypothesized to activate neuronal fibers of central thalamic neurons and *en passant* fibers that broadly innervate the anterior forebrain mesocircuit, including the striatum and frontal cortex, that supports cognition during wakefulness [12].

In this exploratory study, we were able to rapidly validate a promising closed-loop DBS approach by using the flexible and easy to use DyNeuMo-X [7] research platform in a healthy behaving NHP. The internal DyNeuMo system is being used in two clinical trials (NCT05437393, NCT05197816) and the approach developed here demonstrates the safety and feasibly of rapidly testing adaptive CT-DBS protocols – implemented through a single implantable device system – and in future clinical studies, the ability to benchmark our current 12 h–ON and 12h–OFF CT-DBS protocol, which was designed to treat daytime cognitive dysfunction in patients with structural brain injuries [3]. Importantly, daytime CT-DBS in the severely injured brain can have cumulative effects on sleep dynamics that fluctuates over years [13] [14], hence the need for future devices to have real-time clocks and the option of embedded chronotherapy [15]. In addition, future collaborative applications utilizing iterations of this bioelectronic research platform could also expand into the optimization of stimulation parameters across other target nuclei that are important in arousal modulation [16].

## V Limitations And Future Studies

In this study, we present data collected from one animal, implanted unilaterally. Therefore, these results should be considered preliminary and will need to be verified in additional animals that are implanted bilaterally, as is routinely done in the clinical setting for TBI patients. [2], [3]. Standard histology has not been conducted on this animal, therefore the precise locations of each electrode contact are unknown and are currently inferred using our modeling and reconstruction approaches [5], and on the observed behavioral and motor responses to stimulation. The closed-loop experiments are contingent on the animal’s general performance, which is dependent on the animal’s satiety and the difficulty of the task, all of which impact the number of CT-DBS periods across experimental sessions. The DyNeuMo-X was unable to stream signals during closed-loop stimulation, but this was overcome by streaming thalamic LFP signals, along with behavior, to the RZ2 system, to validate changes in the stimulation protocol. In future animals, bilateral ECoG arrays that span larger regions of cortex with more dense sampling (therefore allowing for additional electrophysiological state characterizations), along with bilateral thalamic DBS leads, will be used to further validate the DyNeuMo-X closed-loop CT-DBS approach.

## VI Conclusion

Here we show that automated closed-loop CT-DBS in a healthy NHP can be used to modulate cortical activity and restore behavioral performance. These proof-of-concept results support our goal of developing and accelerating the deployment of novel neuromodulation approaches to monitor and influence the dynamics of arousal regulation; ultimately to treat cognitive dysfunction in patients with structural brain injuries and further etiologies.

## Data Availability

The authors will consider requests to access the data that support the findings of this study in a trusted research environment. Contact: Jonathan L. Baker, job2037@med.cornell.edu.
